# Sensitivity and 3 dB Bandwidth in Single and Series-Connected Tunneling Magnetoresistive Sensors

**DOI:** 10.3390/s16111821

**Published:** 2016-10-31

**Authors:** Michał Dąbek, Piotr Wiśniowski, Tomasz Stobiecki, Jerzy Wrona, Susana Cardoso, Paulo P. Freitas

**Affiliations:** 1 Department of Electronics, AGH University of Science and Technology, Krakow 30-059, Poland; piotr.wisniowski@agh.edu.pl (P.W.); stobieck@agh.edu.pl (T.S.); 2 Silicon Creations, 49 Highway 23 NE, Suwanee 30024, GA, USA; 3 Singulus Technologies AG, Kahl am Main 63796, Germany; jerzy.wrona@singulus.de; 4 INESC-MN and IN, Lisbon 1000-029, Portugal; scardoso@inesc-mn.pt (S.C.); paulo.freitas@inl.int (P.P.F.); 5 Physics Department, Instituto Superior Tecnico, Universidade de Lisboa, Lisbon 1600-276, Portugal; 6 INL-International Iberian Nanotechnology Laboratory, Avenida Mestre José Veiga s/n, Braga 4715-330, Portugal

**Keywords:** current sensors, high-speed electronics, magnetoresistive sensors, sensitivity-bandwidth product, tunneling magnetoresistance

## Abstract

As single tunneling magnetoresistive (TMR) sensor performance in modern high-speed applications is limited by breakdown voltage and saturation of the sensitivity, for higher voltage applications (i.e., compatible to 1.8 V, 3.3 V or 5 V standards) practically only a series connection can be applied. Thus, in this study we focused on sensitivity, 3 dB bandwidth and sensitivity-bandwidth product (SBP) dependence on the DC bias voltage in single and series-connected TMR sensors. We show that, below breakdown voltage, the strong bias influence on sensitivity and the 3 dB frequency of a single sensor results in higher SBP than in a series connection. However, the sensitivity saturation limits the single sensor SBP which, under 1 V, reaches the same level of 2000 MHz∙V/T as in a series connection. Above the single sensor breakdown voltage, linear sensitivity dependence on the bias and the constant 3 dB bandwidth of the series connection enable increasing its SBP up to nearly 10,000 MHz∙V/T under 5 V. Thus, although by tuning bias voltage it is possible to control the sensitivity-bandwidth product, the choice between the single TMR sensor and the series connection is crucial for the optimal performance in the high frequency range.

## 1. Introduction

Since the demand for high-speed current-sensing systems is constantly growing, it is necessary to find new solutions for sensors which can provide the possibility of reliable current monitoring under different conditions. Modern electronic sensor applications have different performance requirements in terms of cost, isolation, bandwidth, measurement precision and range, or size [1,2]. To fulfill these demands, many current measurement methods have been developed over the past years [3]. As the most important features of these methods are related to controlling or monitoring purposes, sensors are required to enable current measurement with high sensitivity and linearity, good thermal stability, low power consumption and within wide frequency range. The ongoing effort in pushing sensors to better performances has led to the development of techniques capable of operating at single electrons, enabling extremely accurate current measurement [4]. However, such sophisticated methods are usually limited to strictly specified conditions, i.e., narrow current range or frequency bandwidth, which significantly reduce their application area.

Nowadays, modern current sensors are expected to provide not only sophisticated but also easily modifiable sensing properties for the highest quality of performance under changeable conditions. Especially when applied in critical, high-speed electronic systems (e.g., power converters), accurate current monitoring, during different, fast changeable operation modes, is the crucial issue to ensure a high level of the system reliability and damage prevention [5,6]. To fulfill these requirements, magnetoresistive (MR) sensors based on magnetic tunnel junctions (MTJ) can potentially be used, providing modification of the sensing properties such as sensitivity or linear range, tunability of the resistance and adjusting dynamic parameters, including frequency bandwidth, which can reach tens of MHz [7,8].

Superior sensing properties [9] make CoFeB/MgO/CoFeB-based tunneling magnetoresistance (TMR) sensors promising candidates for accurate current sensing in a wide frequency range [10]. By connecting the sensors in a bridge configuration, it is possible to reach high sensitivity (tens of mV/V/A) in comparatively wide linear ranges (few mT) [11,12], which can be useful particularly in industrial electronics applications [13]. Additionally, during the fabrication process, sensors can be easily connected in a series, which significantly improves their performance [14]. High 3 dB frequency (3 dB bandwidth), together with high sensitivity, both modulated by bias voltage [15,16], provides the possibility to detect and measure weak signals across a wide frequency range.

We have shown [17] that the 3 dB frequency in single TMR sensors reaches 40 MHz and increases with the DC bias voltage. However, this increase is limited by the breakdown voltage. A similar limitation applies to the sensitivity, which also depends on bias [18]. Thus, single sensors are not suitable for fast, higher voltage applications (i.e., compatible with 1.8 V, 3.3 V or even 5 V standards). These problems can be minimized by connecting sensors in a series. Therefore, establishing the bias voltage influence on the sensitivity and 3 dB bandwidth of CoFeB/MgO/CoFeB-based single and series-connected sensors becomes highly relevant for application in modern, high-speed electronics.

Thus, in this contribution, we have focused on the sensitivity, bandwidth and sensitivity–bandwidth product (SBP) dependence on the DC bias voltage in single and series-connected TMR sensors. We show the nonlinear dependence of sensitivity and SBP on bias voltage in a single sensor, and their saturation below 1 V, and the linear sensitivity and SBP product increase with bias in the series-connected sensors.

## 2. Sensors and Sensitivity—Bandwidth Measurements

Magnetic tunnel junctions based on the CoFeB/MgO/CoFeB structure have been already shown as promising candidates for high-performance magnetic field sensors, both for DC and AC signals. They offer high sensitivity and linearity, modifiable sensing properties and nanosecond dynamic parameters [19]. Sensors with the perpendicular anisotropy (PA) in a sensing layer offer modification of the linear range and sensitivity by PA strength modulation [20]. Moreover, use of the voltage-controlled magnetic anisotropy (VCMA) effect enables control of the linear range, sensitivity and frequency bandwidth via bias voltage strength and polarity [21]. Additionally, by connecting sensors in a series (during the fabrication process), it is possible to shift the breakdown voltage to a higher bias (e.g., 10 V) and improve sensitivity and noise [22,23]. All these properties, together with a low noise level [24,25] and possibility of miniaturization (down to nm), make TMR sensors suitable for modern high-speed sensing systems.

**Figure 1:**
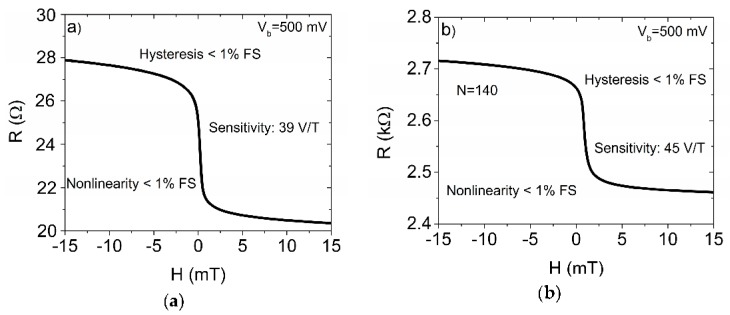
Typical transfer curve of used (**a**) single and (**b**) series-connected sensors (N = 140).

We used single and series-connected (connection of the same single sensors from N = 140 to N = 560) magnetoresistive microsensors based on magnetic tunnel junctions (MTJ) with CoFeB electrodes and perpendicular anisotropy in the sensing layer [9]. The sensors provide high sensitivity, low nonlinearity and hysteresis (Figure 1) [9]. The sensors with the structure Ta(5)/CuN(10)/Ta(3)/PtMn(16)/CoFe(2.3)/Ru(0.85)/Co_60_Fe_20_B_20_(2.3)/MgO(1.3)/Co_60_Fe_20_B_20_(2.3)/Ta(10)/Ru(7) (thickness in nm) were deposited by the TIMARIS magnetron sputtering system at Singulus AG. The wafer was diced into several 1 × 1 in dies. Selected 1 × 1 in dies were patterned using direct write laser lithography and ion beam milling, resulting in circular and square-shaped sensors with areas ranging from hundreds of nm^2^ to over a thousand μm^2^. The patterned dies were annealed in high vacuum at 340 °C for 1 h in a magnetic field of 0.5 T. For this experiment we used square sensors of size 100 × 100 µm with room temperature TMR up to nearly 50%.

**Figure 2:**
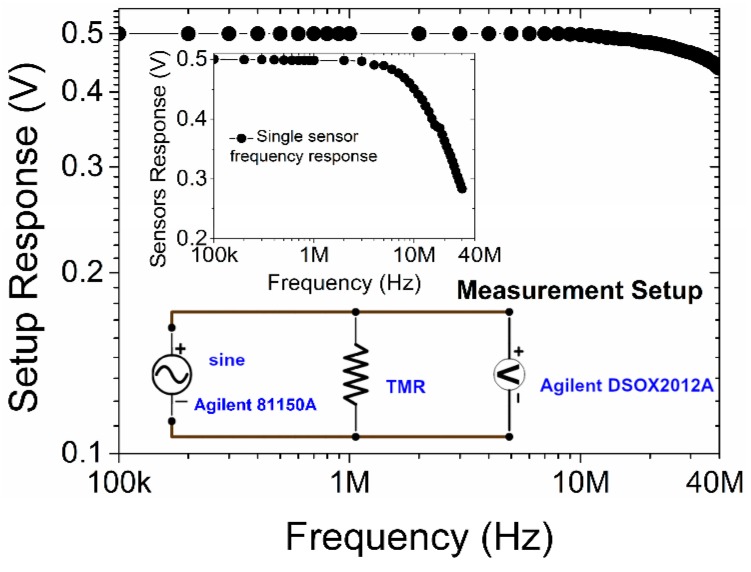
Bandwidth of the measurement system. Inset: Measurement system scheme and frequency response of a single sensor.

To determine the sensitivity and bandwidth of the sensors, we measured the transfer curves (R-H) and frequency response. The transfer curves were measured using a DC four-probe setup with Helmholtz coils and an electromagnet as magnetic field sources. The field sensitivity (FS) was determined as FS = (dR/dH)_max_·I_B_, where I_B_ is the current under the set bias voltage. The 3 dB bandwidth was measured using the Agilent 81150A waveform generator and the Agilent DSOX2012 oscilloscope (Figure 2 inset). We applied a sinusoidal signal of constant peak-to-peak amplitude to the sensors and monitored the voltage on it while scanning the frequency of the signal from 100 kHz to 70 MHz (limit of the setup (Figure 2)). The 3 dB frequency is defined as a frequency at which the monitored signal amplitude of the sensor drops to 0.707 of its initial value (at low frequency). To determine the bandwidth limit of our setup the standard calibration methods (open, short and load) were used.

## 3. Sensitivity—Bandwidth Product

**Figure 3:**
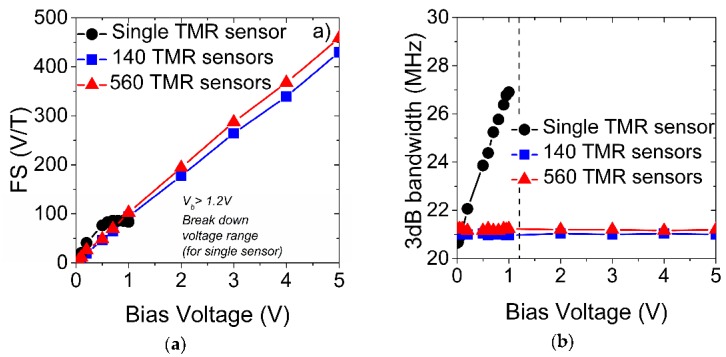
Sensitivity (FS) (**a**) and 3 dB bandwidth (**b**) dependence on bias voltage for single and series-connected sensors.

Only within the low bias range (below 1 V) the sensitivity of a single sensor is higher than in the series connection (Figure 3a). We observe a nonlinear sensitivity dependence on bias in single sensors, which results in its faster increase below 1 V than in a series connection. For series-connected sensors, sensitivity is a linear function of the bias voltage within the whole measured range. Although its increase with the bias is slower than in the single sensor, both sensitivities, of single and series-connected sensors, reach nearly the same value of nearly 100 V/T under 1 V. This is the result of sensitivity saturation in single sensors. In contrast to series sensors for which the sensitivity increases, reaching nearly 450 V/T under 5 V, the saturation of the sensitivity and breakdown voltage limit single sensor performance at a higher bias. Thus, for operation under higher voltage and with high sensitivity, a series connection is required.

The bandwidth of a single sensor increases with the bias while, for series-connected sensors, it is constant in the whole measured range (Figure 3b). Bias voltage does not affect the resistance (and does not have an observable influence on the capacitance) in series connections; thus, the RC product of series-connected sensors is constant in the measured bias range. This is the result of comparatively large numbers of series-connected sensors which minimize the bias influence on the resistance of such a connection. In a single sensor, the bias voltage has a strong influence on the resistance which implicates a significant influence on the RC product. Therefore, compared to a single sensor, where the 3 dB bandwidth (RC product) changes linearly with bias (Figure 3b), the 3 dB frequency of series-connected sensors does not change with the bias voltage (Figure 3b). Additionally, although the resistance of a series connection is N times higher compared to a single sensor, the capacitance of such a connection is N times lower. Thus, the RC product is approximately the same for single and series-connected sensors (under low bias), which indicates that the number of sensors in a series connection does not affect their 3 dB bandwidth (Figure 3b). As a result, the 3 dB bandwidths of single and series-connected sensors (with different Ns) under low bias are similar.

**Figure 4:**
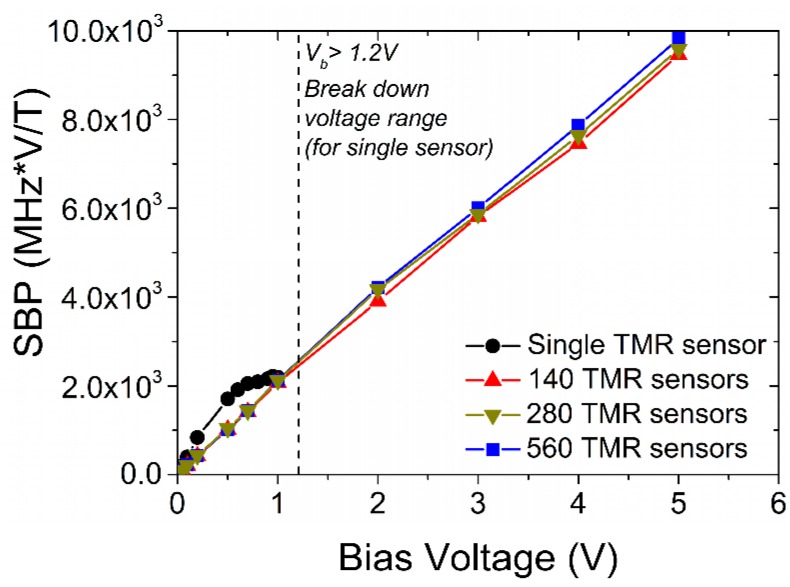
Bias voltage effect on sensitivity-bandwidth product of single and series-connected sensors.

The sensitivity-bandwidth product (SBP) of series sensors increases with the bias voltage and is lower than that for a single sensor only below 1 V bias (Figure 4). The SBP increases faster with the bias for the single sensor because of the nonlinear increase of sensitivity. However, it saturates at about 1 V, reaching nearly the same level as for series-connected sensors of 2000 MHz∙V/T. The higher voltage (above 1 V) performance of a single sensor is limited by the sensitivity saturation and breakdown voltage, which for tested sensors reaches 1.1–1.2 V. For a series connection the SBP increases further with a bias up to nearly 10,000 MHz∙V/T for 5 V (Figure 4). This SBP behavior is mainly the result of sensitivity dependence on the bias voltage, which is nonlinear for the single sensor and linear for the series-connected sensors (Figure 3a). Furthermore, the SBP for series-connected sensors does not depend essentially on the number of sensors (Figure 4), which directly results from the fact that N does not influence the 3 dB bandwidth.

Since the number of series-connected sensors does not have a noticeable influence on the frequency bandwidth, but instead provides the possibility to significantly increase the sensitivity, the series sensor connection is preferred from an application point of view. A large number of sensors in the series connection enable reaching a high SBP, and furthermore, a lower power consumption (high resistance) compared to the single sensor. However, for series-connected sensors a more complex fabrication process is required. Additionally, the series connection has larger area, and thus it can be more difficult to integrate it with other technologies (i.e., CMOS). These features and issues indicate that, although by tuning the bias voltage it is possible to control the sensitivity-bandwidth product, the choice between single and series-connected sensors, with a suitably selected N, is crucial for the optimal performance in a high frequency range.

## 4. Conclusions

We show that by tuning the bias voltage it is possible to control both the sensitivity and bandwidth of single and series-connected TMR sensors. Sensitivity dependence on bias is nonlinear and saturates for single sensors and is linear for series-connected sensors. In the single sensor, the 3 dB bandwidth changes with the bias voltage because it has a strong influence on the sensor resistance. Thus, by tuning the voltage it is possible to control the RC product, which determines the 3 dB frequency of the TMR sensor. In a series connection, when the number of sensors is comparatively large, the voltage influence on the bandwidth is negligible. Additionally, the number of sensors in a series connection does not influence the bandwidth. As a result, the 3 dB bandwidths of single and series-connected sensors (with different Ns) are the same under low bias voltage, but for single sensors it increases significantly with the bias voltage.

The sensitivity-bandwidth product of series sensors increases linearly with the bias voltage, reaching a value of 10,000 MHz∙V/T under 5 V. In contrast, the product for a single sensor increases nonlinearly with the bias and saturates at a value of about 2000 MHz∙V/T under 1 V. As the single sensor performance is limited by the breakdown voltage and saturation of the sensitivity, for higher voltage applications (i.e., compatible to 1.8 V, 3.3 V or 5 V standards) only a series connection can be used, where the SBP increases with the bias. This feature is important especially for modern high-speed electronic applications, where reliable, high-sensitivity sensors become extremely relevant.

The number of series-connected sensors (especially below 100) and the bias voltage influence on the sensitivity-bandwidth product are under further study. The suitable choice between single and series-connected sensors is crucial for optimal performance under different voltage standards in a high frequency range. Considering this, the results of our work can be useful in designing fast and highly sensitive TMR sensors, especially for operating at a bias range above the single sensor breakdown voltage.
